# Supplementary material for: A Likelihood Approach for Real-Time Calibration of Stochastic Compartmental Epidemic Models
Source: PLoS Comput Biol. 2017 Jan 17;13(1):e1005257. doi: 10.1371/journal.pcbi.1005257 (PMC5240920; doi:10.1371/journal.pcbi.1005257)
Supplement: S1 File — (TAR.GZ) [file pcbi.1005257.s014.tar.gz › HSPH_Online-SI-Revision/PF08/n10-extreme/n10-extreme_PF_table0.pdf]

1	R0	{2.68692, 12.5884}	{2.93799, 19.9791}	{{2.88791, 20.}, {2.9973, 20.}}	{9.45411, 58.7105}	inf	2477.	3443.28	{3334.56, 3528.04}	39.0102	0.14279	newly	1264.	1171.83	{1121., 1222.}	7.29193	0.033207	Reff	0.771415	0.9122	{0.861229, 0.994816}	18.2502	ar	9089.	11188.6	{11074., 11295.}	23.1
2	R0	{1.79802, 12.5412}	{2.04327, 19.6573}	{{1.85445, 17.4174}, {2.17193, 20.}}	{13.863, 56.7416}	inf	1208.	1664.71	{1487.09, 1791.06}	38.1095	0.138914	newly	678.	591.91	{542., 638.}	12.7094	0.0599473	Reff	1.00509	1.04477	{0.982883, 1.11225}	5.21947	ar	7412.	9083.88	{8625., 9361.}	22.5564
3	R0	{2.6174, 10.1105}	{2.9685, 17.8637}	{{2.7374, 16.1748}, {3., 18.273}}	{13.4142, 76.6843}	inf	2244.	3245.97	{2993.24, 3321.14}	44.651	0.160068	newly	1325.	1191.53	{1148., 1231.}	10.0732	0.0462717	Reff	0.662726	0.726322	{0.70575, 0.792183}	9.59624	ar	9109.	10729.5	{10510., 10842.}	17.7901
4	R0	{2.27532, 14.3149}	{2.13362, 19.2804}	{{2.04422, 17.1429}, {2.21793, 20.}}	{6.22754, 34.6874}	inf	1860.	1981.82	{1739.51, 2096.42}	9.26187	0.0386707	newly	862.	698.11	{663., 732.}	19.0128	0.0919142	Reff	0.820025	0.872323	{0.813747, 0.935095}	7.07938	ar	8478.	9506.91	{9252., 9706.}	12.1362
5	R0	{2.96649, 18.0674}	{2.79435, 19.7527}	{{2.7486, 20.}, {2.89199, 20.}}	{5.80286, 10.5658}	inf	2876.	3025.23	{2784.19, 3182.03}	8.33974	0.0361876	newly	1043.	1017.61	{944., 1083.}	4.28667	0.019121	Reff	0.663011	0.800597	{0.730923, 0.877014}	20.7518	ar	9476.	10727.2	{10556., 10930.}	13.3143
6	R0	{2.94778, 16.0002}	{2.7393, 19.759}	{{2.63775, 20.}, {2.82138, 20.}}	{7.11536, 23.6814}	inf	2859.	3024.51	{2874.78, 3174.2}	8.01286	0.0339708	newly	1175.	1047.24	{977., 1112.}	10.8732	0.0504979	Reff	0.814766	0.971128	{0.870229, 1.04553}	19.1909	ar	9321.	10431.7	{10210., 10559.}	11.9161
7	R0	{2.17562, 11.7184}	{2.62983, 18.4866}	{{2.20469, 15.604}, {2.94446, 20.}}	{20.9513, 57.7573}	inf	1758.	2790.9	{2191.39, 3152.85}	58.7545	0.196374	newly	1027.	1050.13	{983., 1123.}	4.40019	0.0187715	Reff	0.998827	1.05659	{0.973252, 1.15449}	6.95605	ar	8345.	10343.6	{9830., 10652.}	23.9492
8	R0	{2.7904, 17.8966}	{2.31094, 19.1744}	{{2.1105, 16.9653}, {2.44446, 20.}}	{17.1825, 9.27292}	inf	2748.	2358.75	{2023.61, 2549.34}	14.165	0.0682982	newly	978.	842.19	{800., 879.}	13.8865	0.0652635	Reff	0.871163	0.961879	{0.870815, 1.00788}	10.7846	ar	9285.	10013.1	{9572., 10289.}	7.84136
9	R0	{2.51574, 16.376}	{2.42843, 19.7187}	{{2.22402, 17.8816}, {2.54242, 20.}}	{4.81359, 20.4123}	inf	2361.	2582.59	{2236.41, 2762.12}	11.2845	0.0462396	newly	932.	890.61	{823., 949.}	5.20064	0.0235596	Reff	0.84076	0.895496	{0.791606, 0.978445}	8.13817	ar	8937.	10302.5	{9919., 10542.}	15.2796
10	R0	{2.56405, 18.1787}	{2.18373, 18.3118}	{{2.08315, 17.2056}, {2.37892, 20.}}	{14.8326, 5.80277}	inf	2387.	2095.89	{1968.71, 2370.08}	13.1618	0.0618668	newly	979.	797.69	{714., 866.}	18.5199	0.0900031	Reff	0.996903	1.02635	{0.951419, 1.10307}	5.05657	ar	9005.	9874.9	{9597., 10210.}	9.66019
11	R0	{2.475, 17.1709}	{1.94326, 20.}	{{1.91723, 20.}, {1.96585, 20.}}	{21.4845, 16.4761}	inf	2191.	1705.04	{1666.05, 1744.99}	22.1798	0.10905	newly	841.	593.78	{557., 625.}	29.396	0.151625	Reff	0.935303	0.988976	{0.967864, 1.01044}	5.77358	ar	8870.	9295.44	{9144., 9445.}	4.79775
12	R0	{2.14974, 11.8619}	{2.15043, 18.2646}	{{2.05293, 17.0424}, {2.19046, 20.}}	{2.77866, 53.977}	inf	1788.	2038.4	{1866.4, 2135.01}	14.0439	0.055902	newly	1021.	782.99	{732., 828.}	23.3115	0.116004	Reff	1.0409	1.06261	{1.00021, 1.13683}	4.30706	ar	8414.	9601.97	{9320., 9800.}	14.119
13	R0	{2.20385, 14.4767}	{2.16735, 20.}	{{1.99641, 20.}, {2.25473, 20.}}	{3.29221, 38.153}	inf	1745.	1751.48	{1700.15, 1791.54}	1.74808	0.00760169	newly	848.	595.26	{557., 625.}	29.8042	0.154063	Reff	0.831733	0.877471	{0.826836, 0.951413}	5.83663	ar	8347.	9795.12	{9541., 9969.}	17.349
14	R0	{2.35671, 13.8357}	{2.58886, 19.1196}	{{2.3328, 16.3024}, {2.81516, 20.}}	{12.7195, 40.0991}	inf	2049.	2702.15	{2101.86, 3028.57}	35.24	0.130645	newly	946.	945.14	{827., 1032.}	6.75053	0.0297554	Reff	0.81118	0.840504	{0.742662, 0.925105}	7.60441	ar	8719.	10133.4	{9865., 10413.}	16.2847
15	R0	{1.91368, 10.1082}	{2.15117, 19.5115}	{{2.07274, 17.7505}, {2.22469, 20.}}	{12.5001, 93.0265}	inf	1215.	1992.91	{1842.4, 2118.46}	64.0258	0.213817	newly	838.	698.76	{649., 741.}	16.6158	0.0795862	Reff	0.888904	0.922798	{0.869001, 1.00246}	4.77971	ar	7486.	9600.94	{9413., 9745.}	28.2519
16	R0	{2.48727, 14.882}	{2.75541, 19.9547}	{{2.52596, 20.}, {2.88232, 20.}}	{11.0962, 34.086}	inf	2351.	3198.22	{2875.61, 3368.46}	36.2753	0.133592	newly	1082.	1095.53	{994., 1168.}	5.13956	0.0222934	Reff	0.913574	0.96836	{0.891878, 1.07895}	7.55335	ar	8894.	11024.	{10885., 11172.}	23.9491
17	R0	{2.46795, 13.1915}	{2.84339, 19.2676}	{{2.54458, 17.5577}, {3., 20.}}	{15.9299, 46.0608}	inf	2177.	3127.8	{2684.57, 3487.19}	43.6749	0.155151	newly	1116.	1109.22	{1032., 1190.}	4.69534	0.0205637	Reff	0.942263	0.93891	{0.853472, 1.05909}	7.23329	ar	8796.	10574.4	{10319., 10751.}	20.2183
18	R0	{2.96603, 16.7543}	{2.80714, 19.96}	{{2.75538, 20.}, {2.88092, 20.}}	{5.3569, 19.2143}	inf	2830.	3098.4	{3026.06, 3192.42}	10.1016	0.0420388	newly	1119.	1035.98	{988., 1090.}	7.437	0.033965	Reff	0.741508	0.80438	{0.748271, 0.876061}	8.66248	ar	9394.	10683.9	{10574., 10811.}	13.7315
19	R0	{2.25216, 9.90465}	{2.86114, 19.3}	{{2.4426, 15.7391}, {3., 20.}}	{27.0396, 94.8584}	inf	1991.	3333.84	{2673.67, 3623.21}	67.4453	0.221475	newly	1350.	1194.69	{1132., 1257.}	11.5044	0.0534393	Reff	0.932845	1.0365	{0.907751, 1.18818}	12.6721	ar	8631.	10921.4	{10532., 11148.}	26.5366
20	R0	{2.70837, 18.1923}	{2.39058, 19.6628}	{{2.16691, 18.067}, {2.52434, 20.}}	{11.7335, 9.14611}	inf	2671.	2529.83	{2128.79, 2733.87}	6.45221	0.0301565	newly	986.	882.07	{816., 934.}	10.5406	0.0489707	Reff	0.903241	0.958639	{0.875958, 1.03348}	7.30153	ar	9183.	10173.3	{9780., 10363.}	10.7842
21	R0	{2.30288, 15.5877}	{2.1861, 20.}	{{2.06323, 20.}, {2.2435, 20.}}	{5.0712, 28.3063}	inf	2049.	1838.44	{1778.74, 1892.74}	10.2762	0.047216	newly	836.	636.4	{605., 666.}	23.8756	0.118817	Reff	0.842854	0.976626	{0.922029, 1.01119}	15.8713	ar	8674.	9736.94	{9595., 9873.}	12.2543
22	R0	{2.82208, 10.7544}	{2.64248, 15.7041}	{{2.58849, 14.9871}, {2.67504, 16.2636}}	{7.12072, 46.0246}	inf	2783.	2817.59	{2740.69, 2885.17}	2.51155	0.0103015	newly	1622.	1210.89	{1167., 1249.}	25.3459	0.127088	Reff	0.73233	0.915346	{0.889269, 0.95324}	24.9909	ar	9333.	10175.4	{10079., 10258.}	9.02647
23	R0	{1.69923, 10.1366}	{2.08534, 19.1274}	{{1.84168, 16.9521}, {2.24834, 20.}}	{22.9362, 88.6969}	inf	1080.	1875.61	{1495.88, 2125.78}	74.12	0.237966	newly	667.	677.49	{587., 762.}	8.70915	0.0375125	Reff	0.906029	0.980789	{0.903922, 1.06447}	8.66433	ar	7055.	9315.8	{8701., 9614.}	32.0454
24	R0	{2.64944, 19.054}	{2.16651, 19.8863}	{{2.14139, 20.}, {2.1972, 20.}}	{18.2277, 5.06486}	inf	2322.	2004.45	{1919.32, 2075.48}	13.6756	0.0643809	newly	778.	676.86	{633., 710.}	13.	0.060915	Reff	0.697333	0.818052	{0.78674, 0.836627}	17.3116	ar	9147.	9841.4	{9723., 9965.}	7.59156
25	R0	{2.53191, 10.9214}	{2.46937, 19.9624}	{{2.43455, 20.}, {2.50158, 20.}}	{2.47022, 82.7828}	inf	2411.	2577.86	{2520.1, 2626.59}	7.28599	0.0306	newly	1327.	880.59	{839., 911.}	33.6405	0.178291	Reff	0.740077	0.92051	{0.891691, 0.970472}	24.3802	ar	9009.	10112.3	{10021., 10198.}	12.2464
26	R0	{1.89155, 6.76944}	{2.91393, 19.2159}	{{2.65316, 17.3728}, {3., 20.}}	{54.0498, 183.862}	inf	989.	3216.91	{2705.43, 3452.2}	225.269	0.51045	newly	913.	1116.9	{1044., 1180.}	22.333	0.0870546	Reff	0.863871	0.834438	{0.770085, 0.892722}	5.09857	ar	7348.	10522.8	{10294., 10681.}	43.2069
27	R0	{2.63149, 15.6979}	{2.60907, 19.9696}	{{2.4544, 20.}, {2.78736, 20.}}	{4.95154, 27.212}	inf	2448.	2940.33	{2745.25, 3157.23}	20.2294	0.0792949	newly	1086.	1010.1	{928., 1092.}	7.50645	0.0344728	Reff	0.945758	0.950652	{0.87368, 1.02393}	4.42076	ar	9104.	10567.4	{10403., 10699.}	16.0746
28	R0	{2.85141, 12.6992}	{2.81862, 17.1487}	{{2.68513, 15.5143}, {3., 20.}}	{4.79788, 35.038}	inf	2712.	3136.63	{2930.05, 3515.83}	15.6574	0.0619277	newly	1362.	1218.99	{1133., 1281.}	10.5	0.0485885	Reff	0.727965	0.822283	{0.787885, 0.868706}	13.1563	ar	9317.	10470.9	{10315., 10682.}	12.3848
29	R0	{2.54244, 12.9085}	{2.11845, 18.1817}	{{2.04309, 17.4138}, {2.2113, 20.}}	{16.6765, 40.8506}	inf	2318.	1996.36	{1858.41, 2200.28}	13.8756	0.0657385	newly	1154.	752.94	{718., 784.}	34.7539	0.185715	Reff	0.769342	0.943778	{0.909146, 0.999725}	22.6733	ar	9034.	9611.92	{9413., 9889.}	6.39717
30	R0	{2.10982, 11.7636}	{2.12849, 18.6852}	{{2.05459, 17.1184}, {2.22817, 20.}}	{3.10408, 58.8388}	inf	1626.	2022.69	{1862.95, 2223.43}	24.4243	0.0937976	newly	882.	746.69	{711., 780.}	15.3413	0.0727109	Reff	0.892032	0.968488	{0.90729, 1.04134}	8.57832	ar	8224.	9626.09	{9332., 9917.}	17.0488
31	R0	{2.76312, 15.5396}	{2.70091, 18.9094}	{{2.35036, 15.0992}, {3., 20.}}	{6.08662, 23.7862}	inf	2551.	2925.9	{2395.33, 3245.06}	18.6104	0.0746539	newly	1066.	1043.2	{971., 1105.}	4.04503	0.0179767	Reff	0.809318	0.866343	{0.700866, 0.950029}	11.1028	ar	9220.	10444.9	{9992., 10697.}	13.285
32	R0	{2.21727, 15.5439}	{1.80235, 19.9956}	{{1.73363, 20.}, {1.91435, 20.}}	{18.713, 28.6394}	inf	1810.	1417.62	{1324.1, 1497.09}	21.6786	0.106517	newly	784.	482.96	{452., 521.}	38.398	0.211053	Reff	0.888682	0.923189	{0.814577, 0.977512}	6.87463	ar	8359.	9009.46	{8726., 9409.}	7.78155
33	R0	{2.72912, 10.8159}	{3., 20.}	{{3., 20.}, {3., 20.}}	{9.92554, 84.913}	inf	2166.	3338.31	{3266.65, 3414.11}	54.1233	0.187806	newly	1179.	1086.71	{1042., 1136.}	7.87023	0.0358278	Reff	0.537091	0.624422	{0.596194, 0.655618}	16.26	ar	9196.	11153.3	{11025., 11274.}	21.2845
34	R0	{2.20928, 10.1308}	{2.9399, 19.0371}	{{2.71602, 16.3164}, {3., 20.}}	{33.0705, 87.9134}	inf	1753.	3335.74	{3116.06, 3512.05}	90.2877	0.27755	newly	1080.	1165.7	{1116., 1206.}	7.93889	0.0329382	Reff	0.754911	0.773035	{0.699242, 0.819807}	5.33557	ar	8388.	10594.5	{10437., 10731.}	26.3057
35	R0	{2.10103, 15.2673}	{2.15148, 18.6748}	{{2.05994, 17.1551}, {2.31124, 20.}}	{4.05681, 22.319}	inf	1737.	1992.4	{1859.28, 2148.8}	16.0015	0.0644654	newly	782.	732.61	{659., 789.}	6.75064	0.0313295	Reff	0.894198	0.93666	{0.875185, 1.01347}	5.83217	ar	8264.	9701.82	{9412., 10012.}	17.3986
36	R0	{2.74914, 19.2775}	{2.21676, 19.9433}	{{2.07608, 20.}, {2.27254, 20.}}	{19.3654, 3.96683}	inf	2701.	1948.24	{1889.58, 2013.78}	27.8695	0.142536	newly	946.	671.67	{637., 708.}	28.9989	0.149196	Reff	0.859106	0.945713	{0.889256, 0.988842}	10.0944	ar	9301.	10005.8	{9870., 10175.}	7.77637
37	R0	{2.78161, 16.0136}	{2.56728, 19.9318}	{{2.4624, 20.}, {2.79687, 20.}}	{7.95371, 24.4679}	inf	2648.	2766.89	{2642.9, 2929.39}	5.46262	0.02311	newly	1097.	940.64	{889., 992.}	14.2534	0.0671973	Reff	0.778294	0.884685	{0.737848, 0.968661}	16.1294	ar	9240.	10673.4	{10515., 10828.}	15.5133
38	R0	{2.4454, 8.33501}	{3., 20.}	{{3., 20.}, {3., 20.}}	{22.6793, 139.952}	inf	1846.	3755.15	{3662.62, 3833.99}	103.421	0.30832	newly	1286.	1253.66	{1189., 1307.}	3.3577	0.0149336	Reff	0.652922	0.817	{0.784127, 0.833119}	25.1299	ar	8853.	11310.7	{11116., 11451.}	27.7615
39	R0	{2.9154, 12.3861}	{2.97928, 19.6571}	{{2.98506, 17.5897}, {3., 20.}}	{3.25158, 58.7027}	inf	2561.	3265.32	{2988.74, 3398.92}	27.7133	0.105545	newly	1272.	1098.72	{1047., 1152.}	13.6226	0.0638887	Reff	0.603196	0.770006	{0.729625, 0.817217}	27.6543	ar	9371.	10502.4	{10371., 10614.}	12.0729
40	R0	{2.40923, 15.1963}	{2.36198, 19.539}	{{2.16032, 17.692}, {2.52324, 20.}}	{4.13499, 28.5774}	inf	2019.	2511.94	{2192.99, 2717.83}	24.415	0.093462	newly	887.	879.56	{822., 926.}	3.83991	0.0169456	Reff	0.893824	0.920789	{0.846882, 1.02283}	6.35658	ar	8735.	10294.5	{9903., 10540.}	17.8533
41	R0	{2.63137, 15.238}	{2.19251, 18.1107}	{{2.09178, 16.9871}, {2.31082, 20.}}	{16.6779, 18.8522}	inf	2300.	2067.95	{1880.84, 2306.72}	10.6229	0.0497067	newly	964.	767.68	{726., 802.}	20.3651	0.0992194	Reff	0.798621	0.849906	{0.786061, 0.883549}	8.40412	ar	9150.	9653.85	{9395., 10000.}	5.50656
42	R0	{2.48769, 19.4593}	{1.96544, 19.9914}	{{1.89188, 20.}, {2.00803, 20.}}	{20.9932, 2.73463}	inf	2205.	1570.94	{1457.14, 1629.62}	28.7556	0.147593	newly	727.	531.23	{486., 560.}	26.9285	0.136819	Reff	0.77392	0.849682	{0.809945, 0.934367}	9.78933	ar	8932.	9728.17	{9455., 9892.}	8.91368
43	R0	{2.61199, 10.214}	{2.97828, 19.7549}	{{3., 20.}, {3., 20.}}	{14.0233, 93.4102}	inf	2530.	3359.18	{3333.19, 3431.15}	32.7738	0.122597	newly	1603.	1180.23	{1138., 1224.}	26.3737	0.133155	Reff	0.846024	1.05765	{0.935401, 1.11476}	25.0145	ar	9061.	10501.4	{10404., 10593.}	15.8965
44	R0	{2.50994, 18.3382}	{1.93232, 19.9919}	{{1.90136, 20.}, {1.95873, 20.}}	{23.0133, 9.01782}	inf	2193.	1676.95	{1616.85, 1732.25}	23.5317	0.116806	newly	810.	579.25	{547., 611.}	28.4877	0.146164	Reff	0.937463	0.969805	{0.871035, 1.01524}	5.5563	ar	8827.	9305.81	{9126., 9475.}	5.42438
45	R0	{2.44283, 16.8338}	{2.22135, 19.0338}	{{2.0851, 17.5685}, {2.35405, 20.}}	{9.06665, 13.069}	inf	2140.	2237.89	{2024.84, 2433.48}	7.26176	0.0302521	newly	873.	802.03	{759., 840.}	8.12944	0.037197	Reff	0.849616	0.928739	{0.855534, 0.993026}	9.35401	ar	8878.	10150.7	{9870., 10426.}	14.3357
46	R0	{2.51815, 18.1462}	{2.19702, 20.}	{{1.99839, 20.}, {2.27201, 20.}}	{12.7526, 10.2159}	inf	2349.	1891.72	{1784.14, 1978.49}	19.467	0.09444	newly	861.	649.91	{604., 695.}	24.5168	0.122747	Reff	0.909556	0.951422	{0.874539, 1.01263}	6.12506	ar	8978.	9915.61	{9597., 10101.}	10.4434
47	R0	{2.88885, 16.8987}	{2.10049, 18.622}	{{2.01236, 17.1334}, {2.19158, 20.}}	{27.2899, 10.1976}	inf	2783.	1971.15	{1793.59, 2150.06}	29.1717	0.150851	newly	1104.	734.49	{702., 772.}	33.4701	0.177262	Reff	0.890344	1.00177	{0.961031, 1.07684}	12.5155	ar	9337.	9428.37	{9166., 9676.}	1.87501
48	R0	{2.58709, 13.8786}	{2.83027, 19.842}	{{2.7932, 20.}, {2.91231, 20.}}	{10.4032, 42.9686}	inf	2523.	3250.59	{3171.91, 3394.59}	29.6728	0.112775	newly	1180.	1124.87	{1056., 1183.}	5.11271	0.0231039	Reff	0.854775	0.971384	{0.845769, 1.03635}	14.1985	ar	9146.	10725.	{10615., 10837.}	17.2645
49	R0	{2.42959, 10.7132}	{2.98469, 19.883}	{{3., 20.}, {3., 20.}}	{23.0752, 85.5935}	inf	2209.	3595.08	{3553.67, 3665.49}	62.7468	0.210855	newly	1386.	1218.12	{1177., 1255.}	12.1126	0.0562	Reff	0.872223	0.872746	{0.832245, 0.91218}	3.93472	ar	8878.	10978.3	{10841., 11107.}	23.8666
50	R0	{2.60513, 13.2796}	{2.72591, 19.997}	{{2.6878, 20.}, {2.78025, 20.}}	{5.27284, 50.5846}	inf	2476.	3015.51	{2972.24, 3073.68}	21.7896	0.0854058	newly	1231.	1039.69	{979., 1084.}	15.541	0.0736481	Reff	0.977184	0.981724	{0.95711, 1.00618}	1.98337	ar	9095.	10537.5	{10445., 10648.}	15.8599
